# Double negative T cells (CD4^-^/CD8^-^) are associated with *Trypanosoma cruzi* persistence in the mouse colon during chronic Chagas disease

**DOI:** 10.3389/fimmu.2026.1761769

**Published:** 2026-03-18

**Authors:** Jung-Sun Cho, Supriya Kumar, Erica Silberstein, Xuefei Ma, Alexander Zhovmer, Alain Debrabant

**Affiliations:** 1 Laboratory of Emerging Pathogens, Division of Emerging and Transfusion Transmitted Diseases, Office of Blood Research and Review, Center for Biologics Evaluation and Research, Food and Drug Administration, Silver Spring, MD, United States; 2 Laboratory of Immunobiochemistry, Division of Bacterial, Parasitic & Allergenic Products, Office of Vaccines Research and Review, Center for Biologics Evaluation and Research, Food and Drug Administration, Silver Spring, MD, United States

**Keywords:** Chagas disease, colon, inflammatory DN T cells, regulatory DN T cells, double-negative (DN) T cells, parasite persistence, *Trypanosoma cruzi*

## Abstract

**Introduction:**

Chagas disease (CD), caused by the blood borne parasite *Trypanosoma cruzi*, affects 6–8 million people primarily in Latin America. Chronic CD leads to progressive damage in major organs, particularly the heart and gastrointestinal tract, with significant morbidity and mortality. Despite the gut serving as a key parasite reservoir, the immune response within this tissue during chronic infection remains poorly understood. This study investigates the role of double-negative (DN; CD3^+^/CD4^-^/CD8^-^) T cells in gut immunity during *T. cruzi* infection. Understanding their function could provide insight into mechanisms of parasite persistence and immune regulation in the gastrointestinal tract.

**Methods:**

C57BL/6 mice were infected with a transgenic strain of *T. cruzi* Colombiana expressing a luminescence reporter. We collected tissues from infected mice during the acute (~30 days) and chronic phase (~90 days) of the disease and analyze them by bioluminescence imaging. We isolated cells from the gut lamina propria to identify the major immune cell types by multiparameter spectral flow cytometry. Colon tissue was also analyzed by fluorescence microscopy.

**Results:**

We showed that DN T cells were the major lymphocyte population in this tissue by flow cytometry and fluorescence microscopy. The number of DN T cells increased significantly (~1.5 fold) during the acute phase and remained above control levels during the chronic infection. Two major subtypes were identified: (1) DN T cells with inflammatory phenotypes, which peaked in the acute phase and declined in the chronic phase but remained above control levels, and (2) DN T cells with regulatory phenotypes, which were elevated in both phases.

**Discussion:**

Our findings highlight the critical role of colonic DN T cells in an experimental *T. cruzi* infection. Our results support a model in which DN T cells with inflammatory phenotypes may contribute to controlling the parasite burden in the gut during a chronic infection, whereas the sustained presence of DN T cells with regulatory phenotypes may help maintaining gut homeostasis but also create an anti-inflammatory niche that facilitates long-term *T. cruzi* persistence in this tissue. A better understanding the immune mechanisms occurring in reservoir tissues is important for developing improved therapies for Chagas disease.

## Introduction

Chagas disease is caused by *Trypanosoma cruzi (T. cruzi)*, a protozoan parasite transmitted mainly by triatomine bugs, commonly referred to as “kissing bugs.” This parasite can also be transmitted vertically and by blood transfusion and organ transplantation (1, 2). Chagas disease affects 6–8 million people worldwide, predominantly in endemic countries of Latin America. However, global migration has led to cases being identified in non-endemic areas such as the United States, Europe, and parts of Asia (1). The disease manifests first by an acute phase which is typically asymptomatic or mild, lasting several weeks or few months, but can include symptoms such as fever, fatigue, or swelling at the site of infection. After the acute phase, untreated Chagas disease progresses to a chronic phase which is divided into two forms: indeterminate and determinate. In the indeterminate form, patients show no symptoms, and the disease can remain latent for decades. In contrast, 20-30% of individuals develop the determinate form, which manifests severe complications, primarily affecting the heart and digestive system (1). Cardiac involvement is the most serious complication, leading to cardiomyopathy, heart failure, arrhythmias, or sudden cardiac death (3). Gastrointestinal involvement may lead to conditions such as megacolon or megaesophagus, which result in swallowing difficulties and severe constipation (4). Despite decades of research, vaccine development has been limited, and the feasibility of vaccination as a viable control strategy remains uncertain.

*T. cruzi* infection triggers a robust host immune response involving both innate and adaptive immunity. Macrophages and dendritic cells are among the first responders following an infection. They phagocytose the parasite, produce cytokines such as tumor necrosis factor-alpha (TNF-α), interleukin-12 (IL-12), and nitric oxide (NO), which promote inflammation and recruit more immune cells (5). During the following host adaptive response, both CD4^+^ and CD8^+^ T cells play crucial roles in controlling the infection but also contribute to disease pathology (6–8). They help coordinate the immune response to stimulate macrophages and other immune cells by secreting cytokines including IFN-γ, which is critical for controlling *T. cruzi* infection and support CD8^+^ T cell cytotoxic activity. CD8^+^ cytotoxic T cells are the main effector cells responsible for limiting parasite burden by recognizing the *T. cruzi*-infected host cells via MHC class I molecules and killing them through perforin and granzyme release (9, 10). CD4^+^ T cells are essential for the development and maintenance of CD8^+^ T cell memory during chronic infections (8, 11). Therefore, CD4^+^ and CD8^+^ T cells play import roles in the balanced host adaptive response to *T. cruzi* infection (8, 12).

Regardless of this strong response, *T. cruzi* persists in the infected host resulting in lifelong chronic Chagas disease. In chronic infections, most parasites reside predominantly in heart and skeletal muscle tissues (13). Recent studies using bioluminescence imaging have highlighted the importance of the gastrointestinal tract, particularly the colon, as a key reservoir for *T. cruzi* during chronic infection (14, 15). While much research has focused on cardiac involvement, less is known about local gut immunity in chronic Chagas disease. Colonic tissue from patients with Chagas disease shows a marked increase in T cell infiltrates (16). In chronically infected mice, the colonic gut exhibit sustained inflammation and a marked infiltration of CD45^+^ leukocytes including CD4^+^ and CD8^+^ cells T cells (17).

Double negative (DN) T cells are a subset of T lymphocytes that lack expression of both CD4 and CD8 co-receptors and express either the γδ or αβ T-cell receptors (TCR) on their surface (18, 19). These cells represent a small population in blood and lymphoid tissues and are more represented in peripheral tissues such as skin, lung and gut (19). DN T cells are major sources of immunoregulatory cytokines associated with protective or pathogenic immune responses in infectious and autoimmune diseases (19). With regards to Chagas disease, DN T cells are major sources of either pro inflammatory cytokines in patients with Chagas cardiomyopathy or anti-inflammatory cytokines in individuals in the indeterminate phase of the disease (20, 21). However, the role of DN T cells in the local gut immunity to *T. cruzi* infection has not been studied. Thus, efforts to identify the role of DN T cells in gastrointestinal CD are crucial for understanding how *T. cruzi* evades the host immune system and persists in this tissue.

In this report, we used multiparametric spectral flow cytometry to profile the major immune cells present in the colon of *T. cruzi* infected mice. We observed a significant increase in the number of DN T cells in infected colons. The majority of DN T cells identified exhibit pro-inflammatory properties and likely involved in controlling parasite burden in the gut. However, a distinct DN T cell subset exhibiting an anti-inflammatory phenotype persists during the chronic phase. We propose these anti-inflammatory DN T cells may contribute to the parasite persistence in this tissue during chronic Chagas disease.

## Materials and methods

### Parasites

The Colombian strain of *Trypanosoma cruzi* expressing either nanoluciferase alone (TcCOL-NLuc) or nanoluciferase fused with red fluorescent protein (RFP) (TcCOL-NLuc-RFP) were used to infect LLC-MK2 cells *in vitro* as previously described (15). The tissue culture trypomastigotes (TCTs) were obtained and counted using a Cellometer K2 Fluorescent Cell Counter, following manufacturer’s instructions (Nexcelom Bioscience, MA). The TcCOL-NLuc-RFP transgenic parasite strain was generated as follows. The coding sequence of NanoLuciferase (NLuc) fused to RFP was codon-optimized for expression in *Trypanosoma* species, synthesized, and cloned into pBEX-v2.0 expression vector using *SalI* and *SpeI* restriction sites (Integrated DNA Technologies, IA). Parasite transfections and selection of recombinant clones were performed as previously described (15). The expression of nanoluciferase activity by this transgenic strain (TcCOL-NLuc-RFP) is illustrated in **Supplementary Figure 3**.

### Mice and infections

All animal experiments were reviewed and approved by the White Oak Animal Care and Use Committee (Animal Study Protocol #2010-03). Six to eight weeks old female C57BL/6 mice were purchased from Charles River Laboratories (Germantown, MD) and were housed in cages under specific pathogen-free conditions and maintained in the FDA/CBER AAALAC-accredited facility under standard environmental conditions for this species. The animal protocol is in full accordance with ‘The guide for the care and use of animals’ as described in the U.S. Public Health Service Policy on Humane Care and Use of Laboratory Animals 2015. Our study reflects the results of 2–3 independent experiments with 3–5 mice per group (control, acute at 30 dpi, chronic at 90 dpi) per experiment. The mice were infected with 1 × 10^4^ TCTs in 0.2 ml saline via intra peritoneal (i.p.) injection. Mice were euthanized under terminal anesthesia using 4% (vol/vol) isoflurane gas in oxygen at 2 experimental endpoints: One month post-infection for the acute model and 3 months post-infection for the chronic model. After euthanasia, the mice were perfused with 1X PBS via the heart. Tissues and organs were collected and processed for *ex-vivo* imaging and/or for immune cell isolation as described below.

### *Ex vivo* bioluminescence imaging

**Figure 1:**
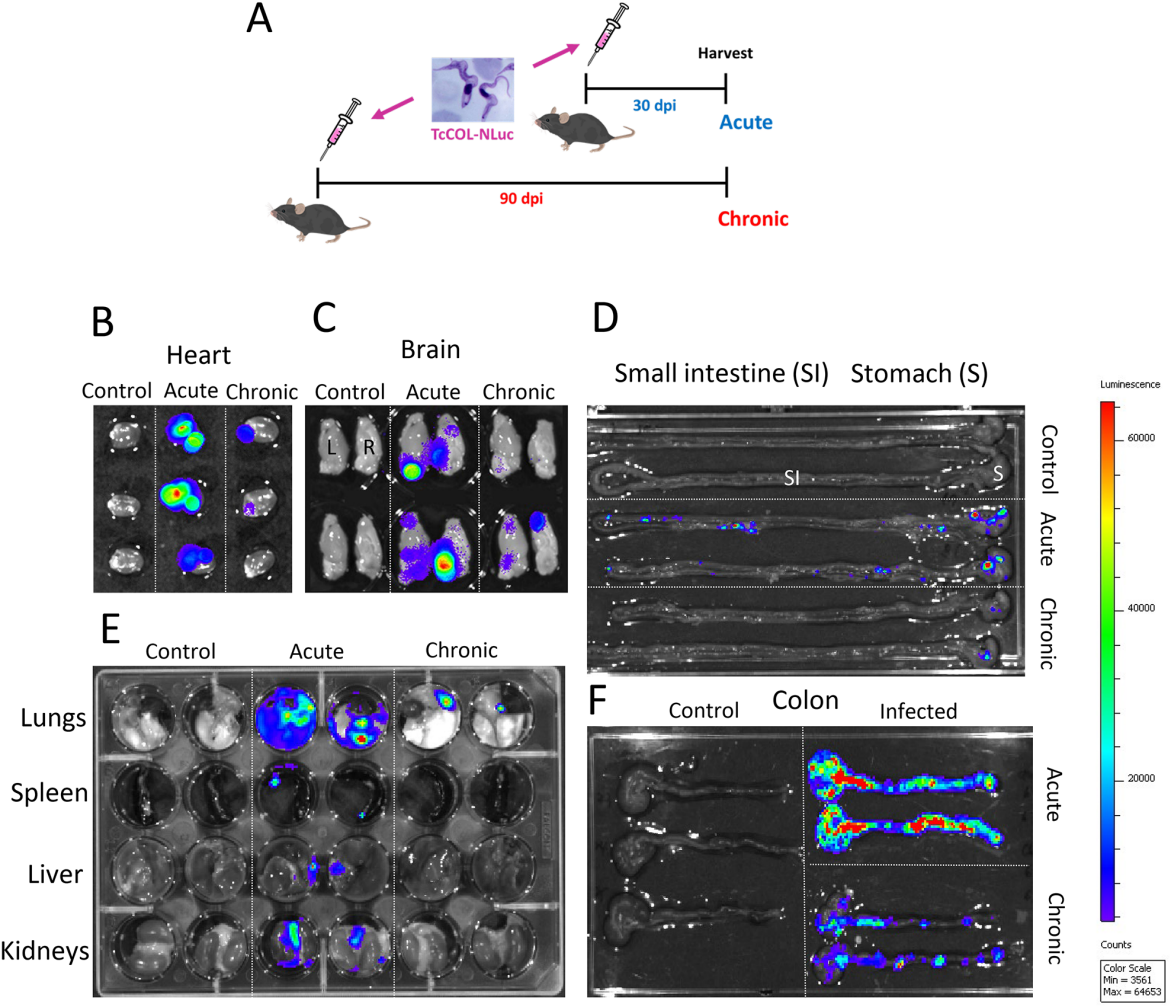
*Ex- vivo* bioluminescence imaging of tissues of *T. cruzi* infected mice. **(A)** Schematic diagram of mice infection. C57BL/6 mice were infected with 10^4^ *T. cruzi* (TcCOL-NLuc) TCTs. Thirty days post infection (dpi), acute phase, and 90 dpi, chronic phase, mice were euthanized, tissues were collected and processed for *ex-vivo* imaging as described in the methods. **(B–F)** *Ex-vivo* bioluminescence images of heart **(B)**, brain **(C)**, stomach and small intestine **(D)**, lung, spleen, liver and kidney **(E)**, and colon tissue **(F)**. Log_10_ heat-map scales represent bioluminescence intensity (blue: low; red: high) expressed in p/sec/cm2/sr. Control: tissues from non-infected mice.

Following euthanasia under terminal anesthesia and perfusion with 1X PBS, the colon, small intestine, heart, lungs, spleen, liver, kidney and brain were excised and subsequently transferred to a dish and soaked in a NanoLuc^®^ Luciferase mixture, prepared by combining one volume of Nano-Glo^®^ Luciferase Assay Substrate with 25 volumes of Nano-Glo^®^ Luciferase Assay Buffer and 25 volumes of 1X PBS. The organs were then imaged using the IVIS Lumina Imaging System^®^ (Caliper Life Science, MA). The exposure time for the images shown in **Figures 1B–F**, was between 10 second to 1 minute depending on individual organs.

### Isolation of lamina propria cells

Following euthanasia, the colon was removed by cutting below the small intestine and above the rectum and transferred immediately onto laboratory tissue paper soaked with 1X PBS. External fat tissue was removed and intestinal content expelled out. Following thorough flushing with 1X PBS to remove the remaining residual material, curved forceps were used to gently run down the length of the colon to squeeze out any remaining mucus. The colon was then cut longitudinally and washed twice by placing it into ice-cold 1X PBS. Remaining mucus was removed by gently scrubbing the tissue on the wet paper towel. The cleaned colon was cut into 0.5 cm pieces and transferred to a 50 ml centrifuge tube containing 20 ml of Buffer 1 (1× HBSS without Ca^2+^ and Mg^2+^ containing 10 mM HEPES, 5 mM EDTA, 5% fetal bovine serum (FBS) and 1 mM DTT). The pieces were incubated for 20 minutes at 37 °C under continuous shaking at 200 rpm using an orbital shaker (Stuart, Orbital Incubator SI500, CA). Then, the pieces were vortexed for 10 seconds and drained onto a 70 µm cell strainer and transferred into a new 50 ml tube containing 20 ml of Buffer 1. The washing step with Buffer 1 was repeated once. Subsequently, the tissue pieces were washed with 1× HBSS without Ca^2+^ and Mg^2+^ containing 10 mM HEPES for 20 min incubation at 37 °C using a shaking incubator. The washed tissue pieces were chopped mechanically using scissors and placed in a gentleMACS C Tube (Miltenyi, MD) containing 5 ml of 1× HBSS with Ca^2+^ and Mg^2+^ containing 10 mM HEPES, 5% FBS, collagenase I (2mg/ml, Invitrogen, CA), collagenase II (2mg/ml, Invitrogen, CA), Dispase (2U/ml, Invitrogen, CA), DNase I (0.5mg/ml, Sigma-Aldrich, MO). The samples were incubated for 30 minutes at 37 °C under orbital shaking and dissociated thoroughly using the gentleMACS Dissociator (Miltenyi, MD) using the instrument intestine program. 20 ml of 1X PBS were added into the digested colon resuspension to stop the enzyme reaction. Each sample was then filtered through a 70 μm nylon mesh filter and the filtrate was centrifuged at 400 g for 5 min to pellet the isolated cells. The pellets were gently resuspended in 20 ml of 1X PBS and filtered through a 30 μm nylon mesh filter and spun down as previously. The supernatants were discarded, and the pellets were gently resuspended in FACS buffer. The cell suspension was counted using a Cellometer K2 Fluorescent Cell Counter, following manufacturer’s instructions (Nexcelom Bioscience, MA).

### Flow cytometry cell staining and processing

The isolated immune cells were resuspended in 1X PBS. Cells were incubated with viability staining using Fixable Viability Stain 510 (BD Biosciences) for 15 min at room temperature protected from light according to manufacturer’s protocol. Cells were fixed with 4% paraformaldehyde in PBS for 10 min, washed with 1X PBS and resuspended in Stain Buffer (BD Biosciences, CA). Fc receptors (FcγR) were blocked with anti-CD16/CD32 antibody (BD Biosciences, CA) for 10 minutes, following manufacturer’s instructions. Cells in 50 µl FACS buffer were used for antibody staining. The antibody Panel 1 (CD45, CD31, PDGFRα and CDH1) was used to characterize immune and non-immune cells. The antibody Panel 2 (CD45, CD3, CD4, CD8, CD11b and B220) was used to profile the immune cells. For analysis of DN T cells, LPC were stained with the antibody Panel 3 including CD45, CD3, CD4, CD8, CCR5, CXCR3, Granzyme B, CCR4, IL10R and IL-10 (**Supplementary Table 1**). The antibody cocktail in Stain Buffer (BD Biosciences, CA) in a total volume of 50 µl was added to the cells and incubated for 60 min at room temperature. For intracellular staining to analyze DN T cell profile, cells were permeabilized and stained for intracellular targets using Granzyme B and IL-10 antibodies in the kit’s permeabilization buffer BD Cytofix/Cytoperm™ (BD Biosciences, CA). For TCR characterization, colonic lamina propria cells were stained with antibodies against CD45, CD3, CD4, CD8, TCRβ, and TCRγδ (see **Supplementary Table 1** for antibody details). Cells were gated on single cells, live, CD45^+^, CD3^+^, CD4^-^, CD8^-^ events and subsequently analyzed for TCRβ and TCRγδ expression. After incubation, cells were analyzed on a Cytek Aurora Flow Cytometer (Cytek Biosciences, CA). Antibodies and reagents were purchased from BD Biosciences, BioLegend, Miltenyi Biotech, R and D Systems, Novus Biologicals, or Thermo Fisher Scientific (**Supplementary Table 1**).

### Flow cytometry reference controls

To prepare reference bead controls of single-stain beads (UltraComp eBeads™, Thermo Fisher Scientific, CA), 20 µl of beads was diluted with 50 µl of Stain Buffer (BD Biosciences, CA) and 1 µl of each antibody was added. One million cells were used for individual fluorescence-minus-one (FMO) controls to develop thresholds for positive/negative marker expression and formally exclude potential spreading-error problems for markers. All controls underwent the exact same protocol as fully stained samples, including washes, buffers used, fixation and incubation steps. The antibodies used to prepare the reference controls are listed in **Supplementary Table 1**.

### Data acquisition and manual analysis of spectral flow cytometry data

Three million cells were analyzed per sample for characterization of the lamina propria (**Supplementary Table 1**, Panel 1) from live-cell population, and for immune cell profiling and DN T cell profiling (**Supplementary Table 1**, Panels 2 and 3) from CD45^+^ gating using a Cytek Aurora Flow Cytometer (Cytek Biosciences, CA). Data were acquired and unmixed using SpectroFlo^®^ v2.2.0.3 software (Cytek Biosciences, CA). Resulting unmixed fcs files were analyzed using manual gating in FlowJo v10.7 software (BD Biosciences, CA) according to the gating strategy (**Supplementary Figure 1**). Cell populations were identified by multiparametric spectral flow cytometry pipeline and confirmed via traditional gating methods.

### Fluorescence microscopy

Following euthanasia and initial perfusion of with 1X PBS, the colons were collected, removed the feces, washed with 1X PBS and fixed with 4% paraformaldehyde (PFA; Sigma-Aldrich, MO) in PBS for overnight at 4°C. PFA-fixed tissues were then rinsed with PBS, placed in 30% sucrose and followed by cryosectioning using Leica CM1860. The 100 micron-thick sections were stored in 1X PBS with 0.1% sodium azide (Sigma-Aldrich, MO) at 4°C until further processing. For staining, stored tissue samples were permeabilized with 0.1% Triton X100 (Sigma-Aldrich, MO) in 1X PBS with 0.05% Tween 20 (Thermo Fisher Scientific, CA) for 60 minutes and blocked for 60 minutes with 1% BSA (Thermo Fisher Scientific, CA) in 1X PBS with 0.05% Tween 20. All antibodies were diluted in 1% BSA in 1X PBS with 0.05% Tween 20 and 0.1% Azide. The duration of the incubation with antibody solutions was 12–24 hours at room temperature. Labeling with Alexa Fluor–conjugated secondary antibodies (Thermo Fisher Scientific, CA) were performed at their final concentration of 5 μg/ml for the duration of 1 hour in 1% BSA PBS-Tween-Azide at room temperature. To wash out the excess of antibodies, we used three consecutive 20 minutes incubations with 1X PBS with 0.05% Tween 20. We mounted washed samples using ProlongGold Antifade (Thermo Fisher Scientific, CA) and imaged 24 hours after, allowing penetration of mounting solution into the sample. Antibodies were polyclonal goat IgG anti-mouse CD4 antibody (R&D Systems, MN), polyclonal rabbit IgG anti-mouse CD8 antibody (Novus Biologicals, CO), monoclonal Rat IgG2b Alexa Fluor^®^ 647 anti-mouse CD3 Antibody (BioLegend, CA), Donkey anti-Goat IgG (H+L) Cross-Adsorbed Secondary Antibody, Alexa Fluor™ 488 (Thermo Fisher Scientific, CA), Donkey anti-Rabbit IgG (H+L) Highly Cross-Adsorbed Secondary Antibody, Alexa Fluor™ 568 (Thermo Fisher Scientific, CA) (**Supplementary Table 1**). Imaging experiments were performed using Leica DMi8 AFC microscope (Leica, Germany) at ×20 magnification. Analyses were performed using Leica LAS X software (Leica, Germany) and the ImageJ/FIJI.

### Statistical analysis

All bar graphs are displayed as means ± SD. The statistical significance of differences between 2 groups was assessed with an unpaired t test. Comparisons of more than 2 groups were performed by one-way ANOVA with multiple comparison tests. All statistical analysis was performed using GraphPad Prism software version 9.0.0. Statistical significance was accepted where *p* ≤ 0.05 (*p ≤ 0.05, **p ≤ 0.01, ***p ≤ 0.001, ****p ≤ 0.0001).

## Results

### Transgenic *T. cruzi* Colombiana expressing nanoluciferase persists in the gut of C57BL/6 mice

We infected 6–8 weeks old female C57BL/6 mice with the Colombian strain of *Trypanosoma cruzi* expressing nanoluciferase [TcCOL-NLuc] (15). Mice were euthanized, and tissues and organs were collected 30 days post infection to reflect the acute phase of the disease and at 90 days post infection corresponding to a chronic infection (**Figure 1A**). *Ex vivo* bioluminescence imaging analysis showed high *T. cruzi* infection in heart, brain, lung, spleen, liver, kidney, small intestine and colon during the acute phase (**Figures 1B–F**). The parasite burden decreased drastically during the chronic phase in most of the tissues tested, except in the colon where significant bioluminescence signal could still be detected at 90 dpi (**Figure 1F**). These results show the colon represents a major site of parasite persistence in C57BL/6 mice for this transgenic *T. cruzi* Colombiana strain.

### Characterization of lamina propria cells isolated from the *T. cruzi* infected mouse colon

To identify the major cell populations present in the mouse colon, we collected this tissue from C57BL/6 mice infected with TcCOL-NLuc parasites and from control mice and isolated lamina propria cells (LPC) using the protocol described in the materials and methods. Flow cytometry analysis of these cell suspensions using CD45, CDH1, PDGFRα and CD31 antibodies (Antibody Panel 1, **Supplementary Table 1**) showed that ~75% of LPC were CD45^+^ immune cells. The LPC preparations also included ~ 20% of epithelial cells (CDH1^+^), less than 5% of fibroblasts (PDGFRα^+^) and ~1% of endothelial cells (CD31^+^) (**Supplementary Figure 1**). These results show that immune cells represent the major cell population of the of the colonic lamina propria.

### Immune cell profiling of the *T. cruzi* infected mouse colon

**Figure 2:**
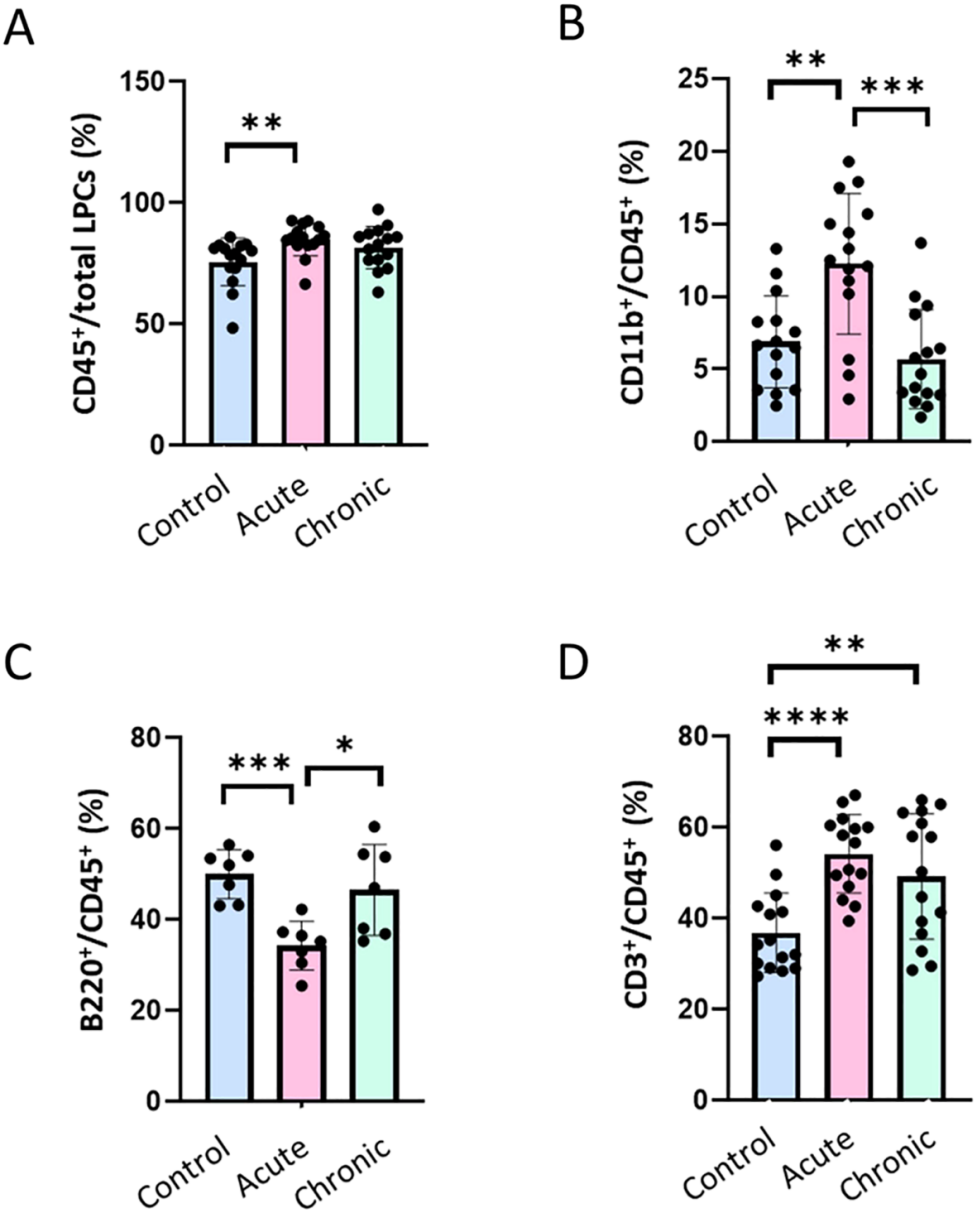
Immune cell composition of the colonic lamina propria during acute and chronic *T. cruzi* infection. C57BL/6 mice were infected with 10^4^ *T. cruzi* (TcCol-Nluc). Mice were euthanized during the acute (30 dpi) and chronic (90 dpi) phases, colon tissues were collected, and single cell suspensions prepared as described in the methods section. Colonic lamina propria cells were analysed by flow cytometry according to the gating strategy shown in **Supplementary Figure 1**. Cells were gated on single cells, live, CD45^+^ events **(A)** and subsequently on **(B)** CD11b^+^ (myeloid), **(C)** B220^+^ (B cells) or **(D)** CD3^+^ (T cell) populations. Bars represent mean ± SD, and individual symbols denote values from single mice (n = 7-15; 3–5 mice per group, 2–3 individual experiments). Statistical comparisons were made by an unpaired t test: *p < 0.05, **p < 0.01, ***p < 0.001, ****p < 0.0001. Uninfected (Control; blue), acutely infected (Acute; pink) and chronically infected (Chronic; green).

To further profile the immune cells in our LPC preparations, cells were labeled with antibodies against surface markers CD45, CD3, CD4, CD8, CD11b and B220 (Antibody Panel 2, **Supplementary Table 1**) and analyzed by flow cytometry following the gating strategy illustrated in **Supplementary Figure 1**. As observed above, the total leukocyte population (CD45^+^) represented 70-80% of total LPCs cells in our preparations and were significantly elevated during the acute phase (p < 0.01; **Figure 2A**). The percent of myeloid cells (CD45^+^, CD11b^+^) increased significantly during the acute phase (~12% of total leukocytes compared to ~6% in non-infected controls) and returned toward baseline levels in a chronic infection (**Figure 2B**). In contrast, the percentage of B cells (CD45^+^, B220^+^) among the total leukocytes was significantly lower in the acute phase compared to non-infected controls (~35% vs. ~50% in controls) and returned to control levels in the chronic phase (**Figure 2C**). Uniquely, T cells (CD45^+^, CD3^+^) increased significantly during acute infection (~55% vs. ~36% of total leukocytes in controls) and remained significantly (p < 0.05) above control levels at the chronic time-point (~50%) (**Figure 2D**). These data suggested a transient myeloid surge, a delayed B-cell recovery, and a sustained elevation of T-cell numbers across the course of *T. cruzi* infection in the colon.

### Multiparametric spectral flow cytometry analysis of myeloid cells, B cells and T cells from the *T. cruzi* infected colon

**Figure 3:**
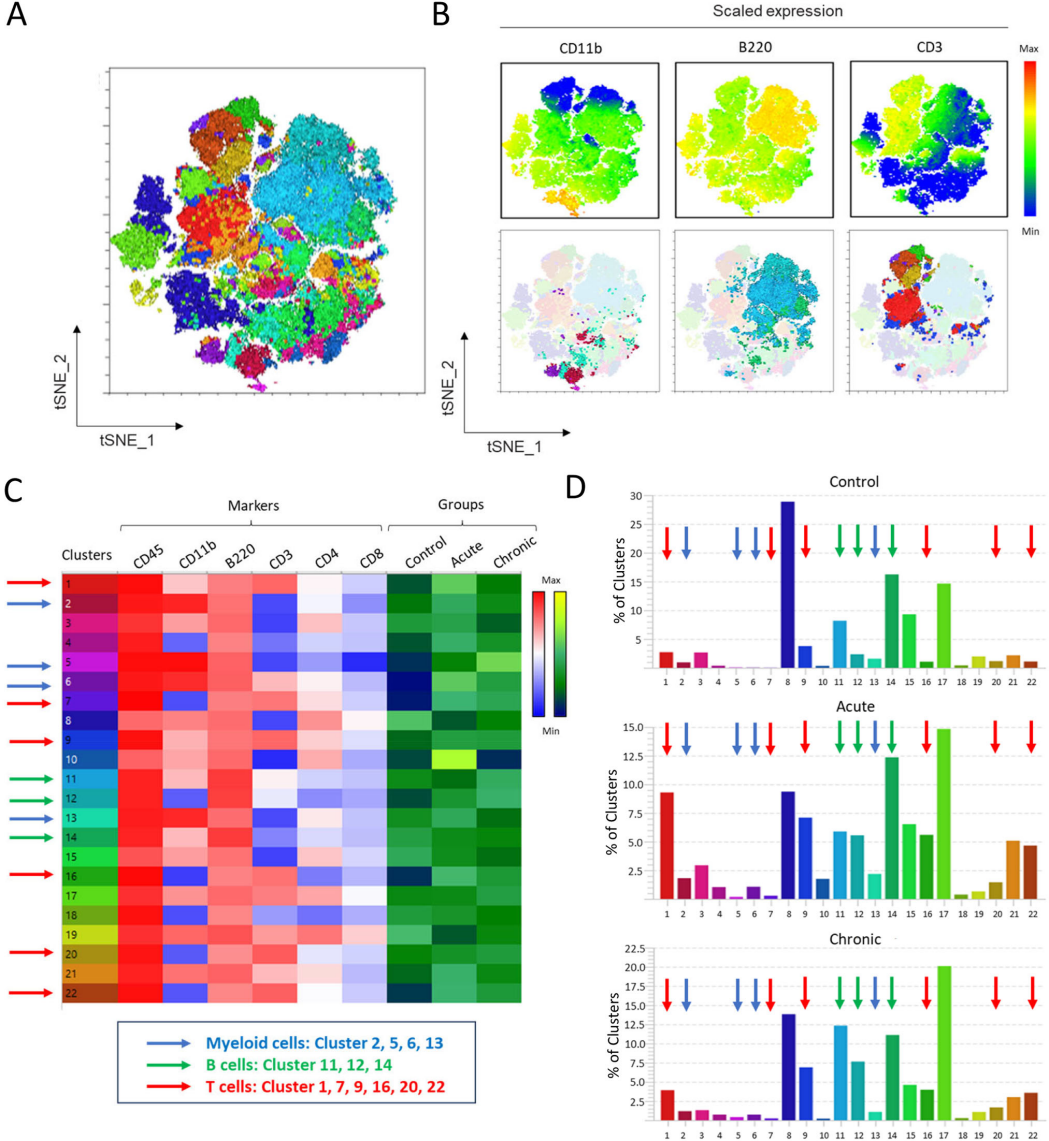
Multiparameter spectral flow cytometry analysis of immune cells of the colonic lamina propria during acute and chronic *T. cruzi* infection. C57BL/6 mice were infected with 10^4^ *T. cruzi* (TcCol-Nluc). Colonic lamina propria cells were isolated from uninfected (Control), acutely infected (Acute, 30 dpi) and chronically infected (Chronic, 90 dpi) mice and analysed by flow cytometry using the gating strategy shown in **Supplementary Figure 1**. **(A)** Automated T-distributed stochastic neighbour embedding (t-SNE) 2D map of the flow cytometry data acquired from control and infected mice colon. **(B)** Upper panel; tSNE 2D map showing scaled expression of CD11b for myeloid cells, B220 for B cells and CD3 for T cells. Lower panel; tSNE 2D map showing the location of CD11b^+^ myeloid cells, B220^+^ B cells and CD3^+^ T cells. **(C)** Heat maps for (left) cell surface markers expression (CD45, CD11b, B220, CD3, CD4 and CD8) and (right) groups (control, acute and chronic colons) for 22 cell clusters identified. **(D)** Percentage of each cluster for each group. Panel **(D)** shows auto-scaled cluster frequencies optimized for visualization within each group. For standardized quantitative comparison across groups, refer to the heat map in panel **(C)**. Arrows in **(C)** and **(D)** indicate immune cell clusters of myeloid cells (blue), B cells (green) or T cells (red).

Multi-parametric spectral flow cytometry is a technique that allows for extensive multicolor panels, enabling simultaneous investigation of many cellular parameters on the surface or in the cytoplasm of cells in a single experiment (22, 23). We used this technique to unbiasedly identify the immune cells present in colon tissue during the acute and chronic infection in our mouse model. We first performed a multiparameter spectral flow cytometry analysis using 6 different surface markers: CD45, CD11b, B220, CD3, CD4 and CD8 (Antibody Panel 2, **Supplementary Table 1**). LPC were analyzed using graph-based clustering analysis (XShift), followed by t-stochastic neighbor embedding (tSNE). **Figure 3A** is a tSNE graphical representation of 22 cell clusters among CD45^+^ immune cells identified in this analysis (clusters shown in **Figure 3C**). **Figure 3B** represents the scaled expression of CD11b for myeloid cells, B220 for B cells and CD3 for T cells (top panels) and show their respective location in the tSNE representation of the data (bottom panels) according to the marker’s expression. The heat map in **Figure 3C** shows the expression level (Blue [low] to Red [high] intensity scale) of each surface marker for the 22 cell clusters within the CD45^+^ leukocytes. The frequency of each of the 22 immune cell clusters is indicated by the Black (low) to Yellow (high) intensity scale for each experimental group (Control, Acute and Chronic). **Figure 3D** shows the percentage of clusters in each experimental group. The scaled expression of CD11b, B220, and CD3 in **Figure 3B** shows that clusters 2, 5, 6 and 13 (Blue arrows) are CD11b^+hi^ and therefore enriched in myeloid cells; clusters 11, 12 and 14 (Green arrows) are B220^+hi^ B-cell populations and clusters 1, 7, 9, 16, 20 and 22 (Red arrows) are CD3^+hi^ T-cell subsets. Stage-specific comparisons revealed a transient expansion of myeloid clusters during the acute phase (30 dpi), which contracted during chronic infection (90 dpi) (**Figures 3C, D**, Blue arrows; **Supplementary Figures 2A–C**). In contrast, the number of cells in the B-cell clusters declined during the acute phase but rebounded to baseline levels in the chronic phase (**Figures 3C, D**, Green arrows; **Supplementary Figures 2D–F**). The T-cell clusters displayed the opposite trajectory, their number of cells increased during acute infection and decreased during the chronic phase **Figures 3C, D**, Red arrows). These unbiased findings are consistent with the manually gated trends shown in **Figures 2B–D** and highlight an infection phase-dependent remodeling of the colonic immune landscape during experimental Chagas disease.

### Multiparametric spectral flow cytometry analysis of the T cell populations in the *T. cruzi* infected colon

**Figure 4:**
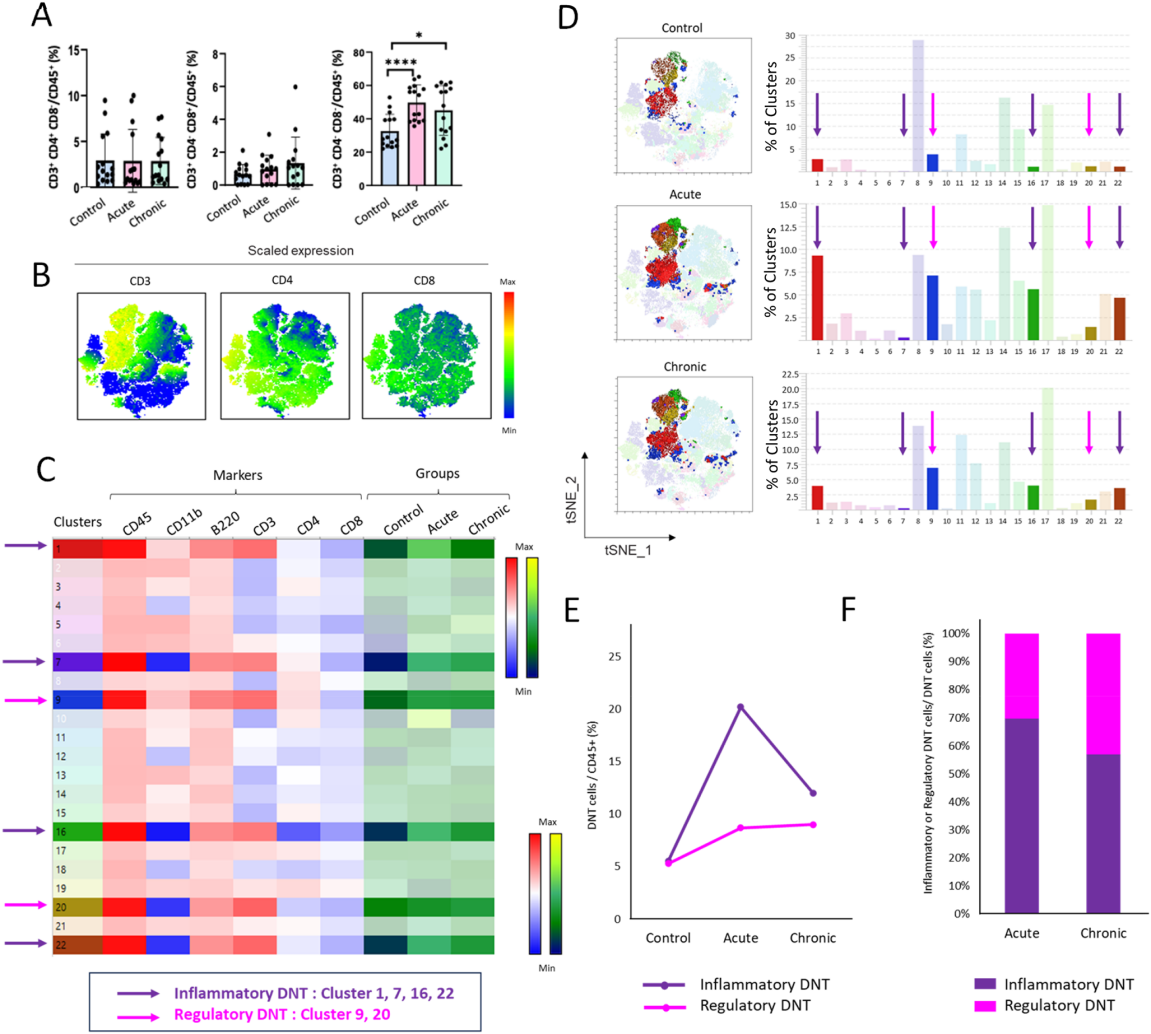
Phenotypic analysis of T cells via multiparameter spectral flow cytometry in the colonic lamina propria during acute and chronic *T. cruzi* infection. C57BL/6 mice were infected with 10^4^ *T. cruzi* (TcCol-Nluc). Colonic lamina propria cells were isolated from uninfected (Control), acutely infected (Acute, 30 dpi) and chronically infected (Chronic, 90 dpi) mice and analysed by flow cytometry using the gating strategy shown in **Supplementary Figure 1**. **(A)** T cells composition by flow cytometry, **(B)** tSNE 2D map showing scaled expression of CD3, CD4, and CD8 cell makers **(C)** Heat map of T cells (CD45^+^, CD3^+^) (highlighted clusters 1, 7, 9, 16, 20, 22). Arrows indicate immune cell clusters of inflammatory (purple) and regulatory (pink) double-negative (DN) T cells. **(D)** tSNE 2D map of DN T cells and percentage of T cells cluster for each group. Arrows indicate DN T cell clusters of inflammatory (purple) and regulatory (pink) cells. **(E)** Percentage of DN T cells with inflammatory/regulatory phenotypes in total immune cells (CD45^+^ cells). **(F)** Relative percentage of DN T cells with inflammatory/regulatory phenotypes in total DN T cells. Panel **(D)** shows auto-scaled cluster frequencies optimized for visualization within each group. For standardized quantitative comparison across groups, refer to the heat map in panel **(C)**. Bars in A represent mean ± SD, and individual symbols denote values from single mice (n = 15; 5 per set, 3 individual sets). Statistical comparisons were made by an unpaired t test: *p < 0.05, ****p < 0.0001.

We sought to elucidate the mechanisms by which *T. cruzi* evades host immunity, persists within colonic tissue, and ultimately drives chronic Chagas disease pathogenesis. Our initial observations indicated that T cells are one of the major immune subsets and their numbers surge significantly (p < 0.05) during the acute phase and remain elevated during the chronic phase compared to uninfected controls (**Figure 2D**). While there was no significant difference between acute and chronic timepoints, both were significantly elevated compared to controls. This biphasic expression pattern suggests a potential link between T cell dynamics and parasite persistence. We therefore focused on the phenotypic and functional characteristics of colonic T cells across the course of infection. To this end, we profiled colonic T cell subsets using traditional and multiparameter spectral flow cytometry using the antibody Panel 3 (**Supplementary Table 1**) and gating strategy shown in **Supplementary Figure 1**. We observed no significant differences in the percentage of conventional CD4^+^ (CD3^+^, CD4^+^, CD8^-^) or CD8^+^ (CD3^+^, CD4^-^, CD8^+^) T cells among total CD 45^+^ cells between uninfected, acute and chronic groups (**Figures 4A**, left and central panels). CD4^+^ and CD8^+^ T cells represented less than 5% of the total CD45^+^ cells in the colon. However, double-negative T (DNT) cells (CD3^+^, CD4^-^, CD8^-^) represented the majority of T cells and showed significant (p < 0.05) expansion during the acute phase, with levels remaining elevated (though not significantly different from acute) during the chronic phase compared to uninfected controls (**Figures 4A**, right panel). Scaled expression of CD3, CD4 and CD8 (**Figure 4B**) and heat map (**Figures 4C**, clusters 1, 7, 9, 16, 20 and 22) confirmed that the majority of CD3^+^ colonic T cells lacked both CD4 and CD8 cell surface co receptors, establishing DN T cells as the predominant T cell subset in the colon.

While DN T cells represented ~30% of colonic leukocytes (CD45^+^) in control mice, their percentage increased to ~50% during the *T. cruzi* acute phase and remained at ~45% in the chronic phase (**Figure 4A**). Further, among the 6 T cell clusters identified in **Figure 3C** (Red arrows), clusters 1, 7, 16, 22 (**Figures 4C, D**, Purple arrows) expanded during the acute phase and declined significantly in chronic infection (Black to Yellow scale), consistent with a transient inflammatory phenotype cell pattern, as inflammatory responses are known to transiently increase during the acute phase and subsequently decrease during the chronic infection (24). In contrast, clusters 9 and 20 (**Figures 4C, D**, Pink arrows) increased during the acute phase and remained elevated during the chronic phase. This pattern is suggestive of regulatory characteristics, as regulatory responses are known to expand during infection and remain elevated into chronic (25). Among the total immune cell population, DN T cells with inflammatory phenotypes exhibited a sharp expansion during the acute phase, followed by a marked decline and recovery during the chronic phase (**Figures 4E**, Purple line). In contrast, DN T cells with regulatory phenotypes increased during the acute phase but remained sustained without decline into the chronic stage (**Figures 4E**, Pink line). Notably, within the total DN T cell compartment in the colon, the proportion of DN T cells with inflammatory phenotypes decreased from ~ 70% in the acute to the ~55% in the chronic phase (**Figures 4F**, Purple bars), whereas the percentage of DN T cells with regulatory phenotypes showed the opposite trend i.e. increasing from ~30% in the acute phase to ~45% in the chronic phase (**Figures 4F**, Pink bars). Collectively, these findings identify DN T cells as the dominant colonic T cell population during *T. cruzi* infection and reveal 2 distinct DN T cells subsets suggestive of inflammatory and regulatory phenotypes.

### Phenotypic validation of DN T cells with either inflammatory or regulatory properties

**Figure 5:**
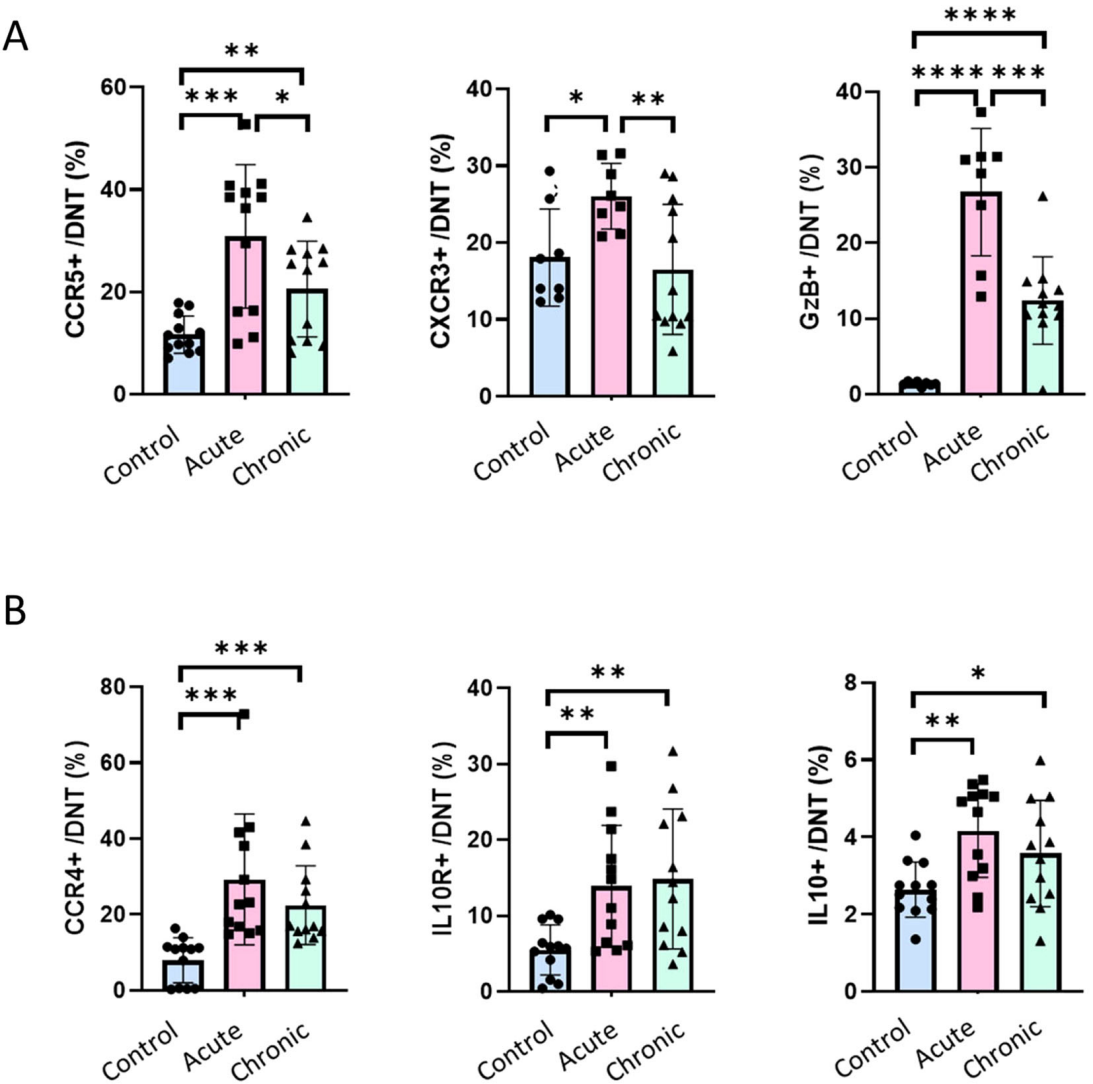
Double-negative T cells phenotypes in the colonic lamina propria of C57BL/6 mice during *T. cruzi* infection. C57BL/6 mice were infected with 10^4^ *T. cruzi* (TcCol-Nluc). Mice were euthanized during the acute (30 dpi) and chronic (90 dpi) phases. Colonic lamina propria cells were isolated from uninfected (Control; blue), acutely infected (Acute; pink) and chronically infected (Chronic; green) mice. Cells were gated on single cells, live, CD45^+^, CD3^+^, CD4^-^, CD8^-^ events and subsequently on **(A)** inflammatory immune cell markers including CCR5, CXCR3 or Granzyme B, and **(B)** regulatory immune cells markers including CCR4, IL10Rα or IL10. Bars represent mean ± SD, and individual symbols denote values from single mice (n = 8-12; 4 per set, 2–3 individual sets). Statistical comparisons were made by an unpaired t test: *p < 0.05, **p < 0.01, ***p < 0.001, ****p ≤ 0.0001.

To support our hypothesis that the DN T cell subset showing a transient increase during the acute phase have inflammatory properties and that the DN T cell subset increasing during the chronic phase have anti-inflammatory/regulatory characteristics, we performed flow cytometry using additional cell surface and intracellular markers (**Supplementary Table 1**, Antibody Panel 3). DN T cells were gated from single cells, live, CD45^+^, CD3^+^, CD4^-^, CD8^-^ events and subsequently on the inflammatory immune cell markers CCR5, CXCR3 and Granzyme B (GzmB), and the regulatory immune cell markers CCR4, IL10Rα and IL10, as illustrate in **Supplementary Figure 1**. As expected, DN T cells expressing inflammatory makers such as CCR5, CXCR3, or GzmB increased sharply during the acute phase and contracted significantly during chronic infection yet remained elevated relative to controls (**Figure 5A**). Also agreeing with previous results (**Figure 4**), DN T cells expressing regulatory makers such as CCR4, IL-10Rα, or IL-10 were significantly expanded in both acute and chronic phases (**Figure 5B**). These results confirm the presence of two distinct DN T cell subsets in the colon of *T. cruzi* infected mice. One subset of DN T cells with inflammatory phenotypes which transiently expands during the acute phase and another subset of DN T cells with regulatory phenotypes which persists in the gut and which proportion increases substantially during the chronic phase (**Figures 4F**, Pink bars).

To determine whether the colonic DN T cells we identified express αβ or γδ T cell receptors, we performed additional flow cytometry analysis using TCRβ and TCRγδ antibodies. In uninfected control mice, TCRβ^+^ cells comprised approximately 87% of total DN T cells, while TCRγδ^+^ cells represented approximately 11% (**Supplementary Figures 4A, B**). During *T. cruzi* infection, the proportion of TCRβ^+^ DN T cells decreased significantly (p < 0.05) to approximately 70% in both acute and chronic phases, while TCRγδ^+^ DN T cells increased modestly but significantly (p < 0.05) to approximately 13% during chronic infection compared to the acute phase (**Supplementary Figures 4A, B**).

### Immuno-staining of DN T cells in the colon of *T. cruzi* chronically infected mice

**Figure 6:**
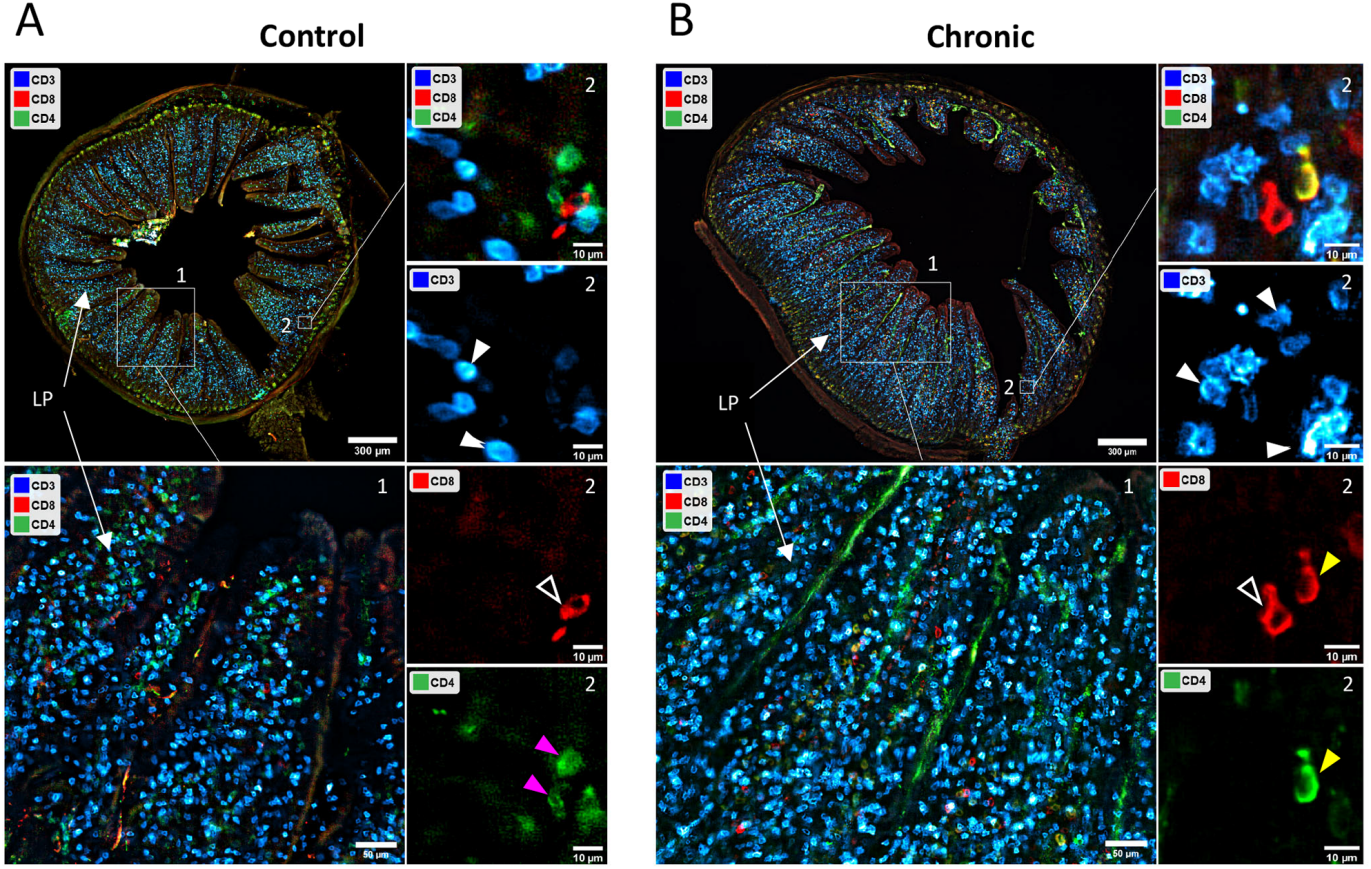
Microscopy analysis of *T. cruzi* infected colons. C57BL/6 mice were infected with 10^4^ *T. cruzi* (TcCol-Nluc-RFP). Colons were isolated from control and chronically infected mice (90 dpi), processed for microscopy and stained with CD3 (Blue), CD4 (Green) and CD8 (Red) specific antibodies (**Supplementary Table 1**, Antibody Panel 4) as described in the materials and methods. Immunofluorescence images of cross sections of colons obtained from non-infected mice (Control) and chronically infected mice (Chronic) are shown in panels **(A, B)**, respectively. In **(A, B)**, a low magnification image of the entire colon is shown in the upper left, a region of interest (ROI) 1 is shown at mid-magnification in the lower left, and a high magnification image of ROI 2 is shown in shown on the right. For ROI 2, an overlay image of the 3 channels CD3 (Blue), CD4 (Green) and CD8 (Red) is shown on the top right, and the individual channels are represented below as indicated. A representative result of 3 controls and 3 infected colons is shown in this figure. LP, Lamina Propria; solid white arrow heads, double-negative T cells (CD3^+^, CD4^-^, CD8^-^); open arrowheads, CD3^-^, CD4^-^, CD8^+^ cells; solid pink arrowheads, CD3^-^, CD4^+^, CD8^-^ cells; solid yellow arrowheads, CD3^-^, CD4^+^, CD8^+^ cells.

To support our flow-cytometric findings that DN T cells comprise the major colonic T cell subset during a *T. cruzi* infection, we stained colon sections obtained from uninfected control and chronically infected mice with CD3, CD4 and CD8 specific antibodies (**Supplementary Table 1**, Antibody Panel 4). At low magnification, the overall colonic architecture was preserved, allowing visualization of mucosal villi crypts, with clear identification of the lamina propria (LP) (**Figures 6A, B**, top left panels). CD3^+^ cells were predominantly distributed within the LP. At mid-magnification, the majority of CD3^+^ T cells lacked both CD4 and CD8 (blue on the merged image) identifying them as DN (CD3^+^, CD4^-^, CD8^-^) T cells. At high magnification, co-localization analysis confirmed that most CD3^+^ T cells were negative for both CD4 and CD8, reinforcing DN T predominance in the colon of both uninfected and chronic infected mice (**Figures 6A, B**, right 4 panels). Less frequently, we observed CD3^-^/CD4^+^; CD3^-^/CD8^+^ and CD3^-^/CD4^+^/CD8^+^ cells within the tissue. Because these cells lack CD3, they are unlikely to represent conventional T cells and may reflect other leukocyte populations resident in the colon. Collectively, these spatially resolved images support our multiparameter spectral flow cytometry data (**Figure 4**) showing that the majority of colonic CD3^+^ cells are CD4^-^ and CD8^-^ DN T cells.

## Discussion

During the acute phase of *Trypanosoma cruzi* infection, a coordinated innate and adaptive response - dominated by cytotoxic CD8^+^ T cells, IFN-γ-producing CD4^+^ T cells, activated macrophages and NK cells - rapidly reduces parasitemia by lysing extracellular trypomastigotes and killing parasite-infected host cells (7, 11, 26). Although this response clears most parasites, some escape and reside long term in some tissues including the heart, skeletal muscles, skin and gut, contributing to chronic pathology (14, 27). Understanding how the parasite evades immune detection and why the host immune system fails to eradicate these residual foci remains a critical key to curing chronic Chagas disease. To date, most studies have focused on cardiac involvement, leaving a substantial knowledge gap in other tissues that also serve as major parasite reservoirs. Accumulating evidence in animal infection models indicates that the gastrointestinal (GI) tract, especially the colon, is one of the dominant long-term tissue reservoirs for *T. cruzi* in chronic Chagas disease (14, 15). Using bioluminescence and confocal imaging, Lewis and colleagues further showed that persistent parasite nests mostly localize to smooth-muscle cells in the colonic circular muscle layer of chronically infected mice (14, 27). Persistence in the GI tract was reported for diverse *T. cruzi* strains (14, 15, 27). Since little is known about local immunity to *T. cruzi* in the GI track, we directed our investigation toward dissecting the gut immune response and the mechanisms that facilitate parasite persistence within the intestinal niche. As a first step toward addressing these questions, we used flow cytometry to analyze the immune cell composition of *T. cruzi* infected colon in a mouse model of CD.

We first confirmed that the gut was a major site of parasite persistence in the animal model used in these studies, in which C57BL/6 mice are infected with the *T. cruzi* Colombiana strain expressing nanoluciferase [Tc-COL-NLuc (15),] (**Figure 1F**). These results agree with our previous observations that this transgenic parasite line also persisted in the gut of Swiss Webster mice (15). Although both the small and large intestine of chronically infected Swiss Webster mice remained infected, only the colon appears to be the major site of persistence in C57BL/6 mice. Such variability in site of persistence across mouse strains has been reported previously and is likely influenced by host and parasite genetic variability (28). We also observed that the heart, brain and the lungs of C57BL/6 mice still harbored some parasites at 90 dpi (**Figures 1B, C, E**), as observed in other infection models (15, 29, 30).

Having established that the colon was a major site of parasite persistence in our model, we then analyzed the immune cell composition of this tissue by flow cytometry. Our results showed that the majority (70-80%) of the cells were CD45^+^ leukocytes (**Figure 2A**). The percentage of CD 45^+^ leukocytes in LPC isolated from acutely infected colons (30 dpi) was significantly higher than in controls, suggesting infiltration of immune cells in this tissue following a *T. cruzi* infection. This agrees with previous observations showing the infiltration of CD45^+^ cells in colonic tissue after *T. cruzi* infection (17). Among these leukocytes, we detected a significant expansion of CD11b^+^ myeloid cells in acutely infected colons, followed by their decrease to non-infected tissue level during the chronic phase (90 dpi, **Figure 2B**). Although our single-marker strategy cannot distinguish neutrophils, inflammatory monocytes, macrophages or myeloid-derived suppressor cells (MDSCs), this biphasic pattern reflects the effective systemic host immune response during the acute phase, including activation and expansion of myeloid cells followed by their contraction and the return to steady state levels during the chronic phase (31, 32). In contrast to myeloid cells, we observed a significant reduction of the percentage of B220^+^ B cells among total leukocytes in the colon during the acute phase followed by their return to pre-infection levels in the chronic phase (**Figure 2C**). A similar strong depletion of mature B cells was reported in the spleen of infected mice within 3 weeks of infection. This loss originated in the bone marrow and coincided with heightened apoptosis of pro- and pre- B cell populations (33). Of significance, we observed that a large proportion of colonic leukocytes were represented by CD3^+^ lymphocytes (**Figure 2D**). This likely reflects the well-established and effective host cellular immune response to a *T. cruzi* infection (7, 9, 34). Acute *T. cruzi* infection induces a rapid expansion of IFN-γ- and perforin-producing CD8^+^ T cells, which are critical for early parasite control (35). CD4^+^ T lymphocytes are also produced as part of the host response (36). They represent a diverse population of cells involved in coordinating and modulating the immune response and are important for the development and maintenance of CD8^+^ T cell memory during chronic infections (10). In mice models, infiltrated CD4^+^ and CD8^+^ cells were observed in close proximity to parasitized myocytes in chronically infected colons (14). In the gastrointestinal tract, T cell responses help limit parasite burden, yet incomplete or localized immunity may permit ongoing infection and tissue damage. In our study, the number of T cells markedly increased in the colon during the acute phase of *T. cruzi* infection, reflecting robust effector expansion in response to the parasite. Although the percentage of T cells in the chronic phase was not significantly different from the acute phase, T cells remained significantly elevated compared to uninfected controls (**Figure 2D**), suggesting ongoing immune activation during chronic infection.

Given our observation that the CD3^+^ lymphocytes infiltrating the colon remained elevated during the chronic phase, we further analyzed this colonic population using multiparameter spectral flow cytometry. This technique allows for extensive multicolor panels, enabling the simultaneous investigation of numerous cellular parameters, both on the surface and within cells, in a single experiment (22, 23). Using multiparameter spectral flow cytometry, we unbiasedly identified that the majority of CD3^+^ T cells expanding in the colon following *T. cruzi* infection were CD3^+^CD4^-^CD8^-^ double-negative (DN) T cells (**Figures 3**, **4**). Further this was supported by our microscopic observations that DN T cells were the majority population in the colon and located in the lamina propria compartment (**Figure 6**). In general, DN T cells constitute a minority population among all T cells of the immune system, however, they can be an important source of cytokines and chemokines and can have both inflammatory and regulatory functions (18, 19). DN T cells circulate in small numbers in peripheral blood and are enriched in non-lymphoid organs, such as the kidney, lung, liver and intestinal epithelium (19, 37). In Chagas disease, circulating DN T cells include functionally distinct αβ and γδ TCR-expressing subsets, which exhibit differential activation patterns associated with the indeterminate and cardiac forms of the disease (38). More recently, distinct memory DN T cell subpopulations have been identified, with inflammatory memory populations preferentially expanded in Chagas cardiomyopathy patients and implicated in pathological immune activation (39).

Our observation is particularly significant because, although DN T cells have been implicated in systemic immune responses during Chagas disease, their expansion in colon tissues following *T. cruzi* infection has not been described. To our knowledge, this represents the first demonstration that gut-associated DN T cells constitute the predominant T cell population responding to *T. cruzi*. Further, our study revealed distinct phase-dependent dynamics of DN T subsets during *T. cruzi* infection (**Figures 4**, **5**). We characterize two phenotypically and functionally distinct subsets: one displaying inflammatory features expressing CCR5, CXCR3 or Granzyme B, and another with regulatory properties expressing CCR4, IL-10Rα, or IL-10 (**Figure 5**). Specifically, in the acute phase, we observed a marked expansion of both DN T cells with inflammatory/regulatory phenotypes in the colon, suggesting a dual role in parasite clearance and tissue remodeling. In contrast, during the chronic phase, DN T cells with inflammatory phenotypes contracted significantly but remained above control levels, whereas DN T cells with regulatory phenotypes persisted at comparably high frequencies. This sustained regulatory signature highlights a previously unrecognized mechanism by which DN T cells may simultaneously shape tissue repair and promote a permissive niche that is associated with long-term parasite persistence in the gut. This observation reflects the dynamic balance between host immunity and parasite persistence in Chagas disease, where regulatory mechanisms ultimately support the chronic stage of infection.

Despite recent efforts, the origin of colonic DN T cells remains unclear (19). DN T cells may derive from thymus developed from bone marrow progenitors through defined stages. Their presence in peripheral blood may result from their evasion of thymic negative selection, followed by activation and expansion in the periphery (18). Accumulating evidence also suggests that conventional CD4^+^ T cells or CD8^+^ T cells can downregulate the expression of their co-receptor including CD4 or CD8 and transition into a DN T phenotype (19, 40, 41). Our TCR characterization revealed that TCRαβ^+^ DN T cells are the predominant subset (approximately 70-87% of total DN T cells), while TCRγδ^+^ DN T cells represent a minority population (approximately 9-13%) (**Supplementary Figure 4**). The modest but significant increased proportion of TCRγδ^+^ DN T cells during chronic infection suggests they may contribute to the increase in DN T cells with regulatory phenotypes observed in **Figure 5B** since this DN T cell subtype have been associated with anti-inflammatory functions (42). Future studies should determine whether the inflammatory and regulatory phenotypes we identified are differentially distributed between TCRαβ^+^ and TCRγδ^+^ DN T cell subsets. Additional studies will be needed to determine the origin of the DN T cells infiltrating the colon following *T. cruzi* infection.

Our observations are limited to a single mouse/parasite model of Chagas disease, i.e. C57BL/6 infected with *T. cruzi* Colombiana parasites (Discrete Typing Unit 1 (43)). We believe that the expansion of DN T cells in response to *T. cruzi* infection and their contribution in creating a permissive and anti-inflammatory environment that favour long-term parasite survival in the gut is applicable across parasite lineages and mouse strains as other *T. cruzi* lineages/mouse strain combinations resulted in similar parasite persistence in this tissue (28). We analysed immune cells from lamina propria preparations, while parasites preferentially persist in smooth muscle cells of the circular muscle layer (17, 27). Therefore, the immune cells we characterized may not be in direct contact with parasite nests. Our findings reflect the immune landscape of the lamina propria compartment, which may influence but not directly interact with parasitized muscle cells. Future studies should employ spatial transcriptomics to determine whether DN T cells are also located in proximity to parasitized muscle cells and to assess whether local DN T cell phenotypes correlate with parasite burden at the tissue microenvironment level. Our findings are correlative and do not establish causality. While we demonstrate a strong association between DN T cells with regulatory phenotypes and parasite persistence, functional studies including DN T cell depletion, adoptive transfer, or blockade experiments would be required to definitively establish whether these cells directly contribute to parasite survival. Whether our observations can be extrapolated to human infection would require additional studies including direct microscopic evidence from human colon tissue samples.

In summary, our study highlights DN T cells as critical yet previously underrecognized mediators of gut immunity during *T. cruzi* infection. In the acute phase, we observed a marked expansion of DN T cells with inflammatory phenotypes, likely contributing to parasite clearance, accompanied by an increase in DN T cells with regulatory phenotypes which balance inflammation and facilitate tissue remodeling. As the infection progresses to the chronic stage, DN T cells with inflammatory phenotypes cells contracted to near pre-infection levels, whereas DN T cells with regulatory phenotypes persisted. We propose that these long-lasting DN T cells with regulatory phenotypes not only promote tissue repair but also contribute to creating a permissive anti-inflammatory environment that is associated with *T. cruzi*’ s ability to evade the host immune control and establish long-term persistence in the host. Their dual capacity to exert both inflammatory and regulatory functions underscores a central role for DN T cells in balancing host defense with parasite survival. Further elucidation of DN T cells’ biology will be essential to determine their potential as novel therapeutic targets in Chagas disease.

## Data Availability

The original contributions presented in the study are included in the article/**Supplementary Material**. Further inquiries can be directed to the corresponding author.
