# Supplementary material for: Double negative T cells (CD4-/CD8-) are associated with Trypanosoma cruzi persistence in the mouse colon during chronic Chagas disease
Source: Front Immunol. 2026 Mar 18;17:1761769. doi: 10.3389/fimmu.2026.1761769 (PMC13038538; doi:10.3389/fimmu.2026.1761769)
Supplement: Supplementary file 1 [file DataSheet1.pdf]

1. Supplementary Figures

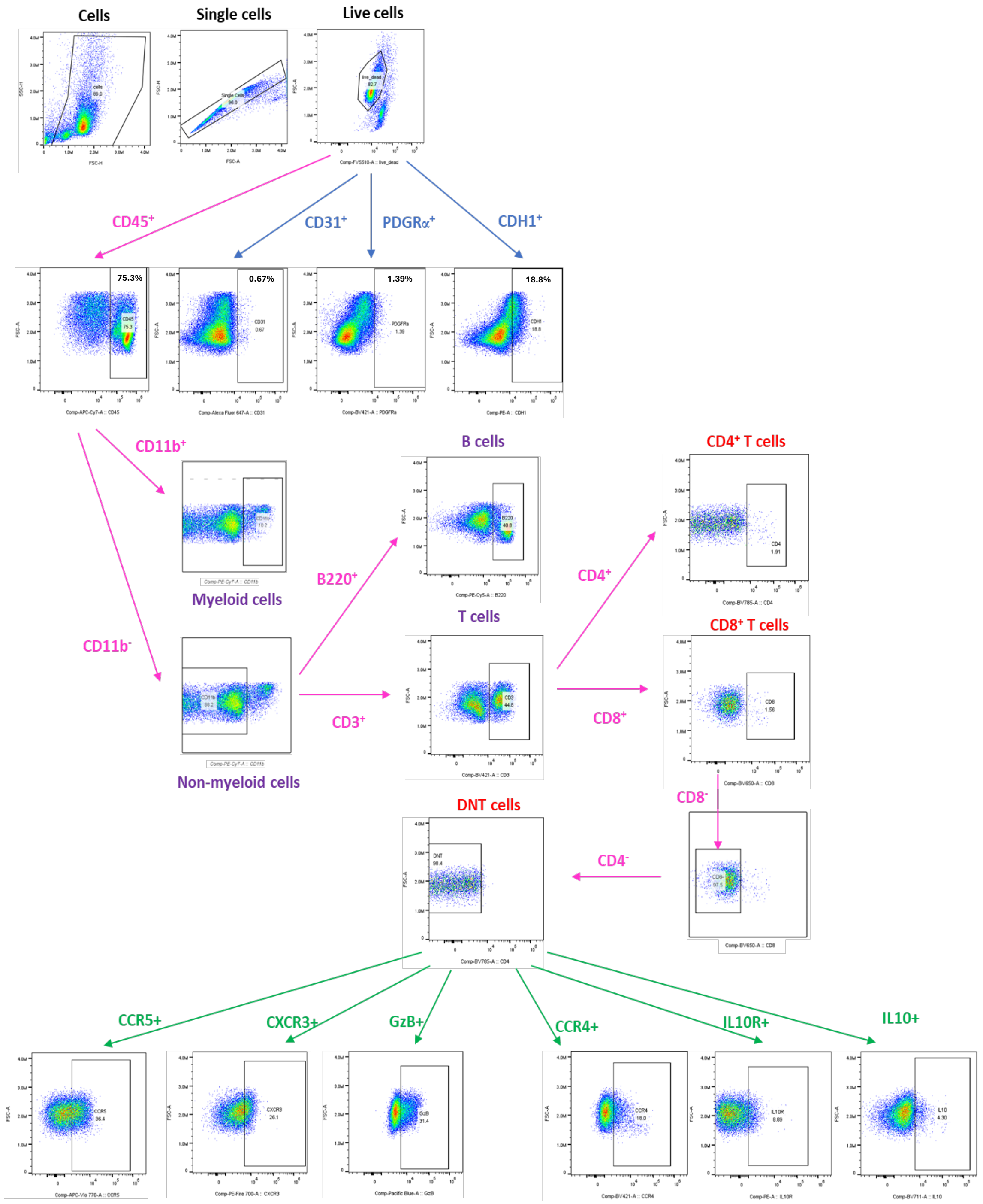

Supplementary Figure 1. Flow cytometric gating strategy for colonic lamina propria cells isolated from C57/B6 mice. For all flow cytometry experiments, cells were first gated on single cells and live cells. For the initial analysis of the colonic lamina propria (Fig.2), cells were

subsequently gated on CD45$^+$ (immune), CD31$^+$ (endothelial), PDGFRα$^+$ (stromal) and CDH1$^+$ (epithelial) cell populations. For the analysis of CD45$^+$ cells (Fig 3), cells were gated on CD11b positive (myeloid) or CD11b negative (lymphoid) cells. Subsequently, B cells, CD4$^+$ and CD8$^+$ T cells, and double-negative (CD4$^-$, CD8$^-$;DN) T cells were identified as indicated. For the analysis of DN T cells (Fig 5), from the total DN T cell population, the following markers were used to identify inflammatory DN T cells (CCR5$^+$, CXCR3$^+$, GzB$^+$) and regulatory DN T cells (CCR4$^+$, IL10R$^+$, IL10$^+$). SSC-H: side scatter height, FSC-H: forward scatter height, FSC-A: forward scatter area.

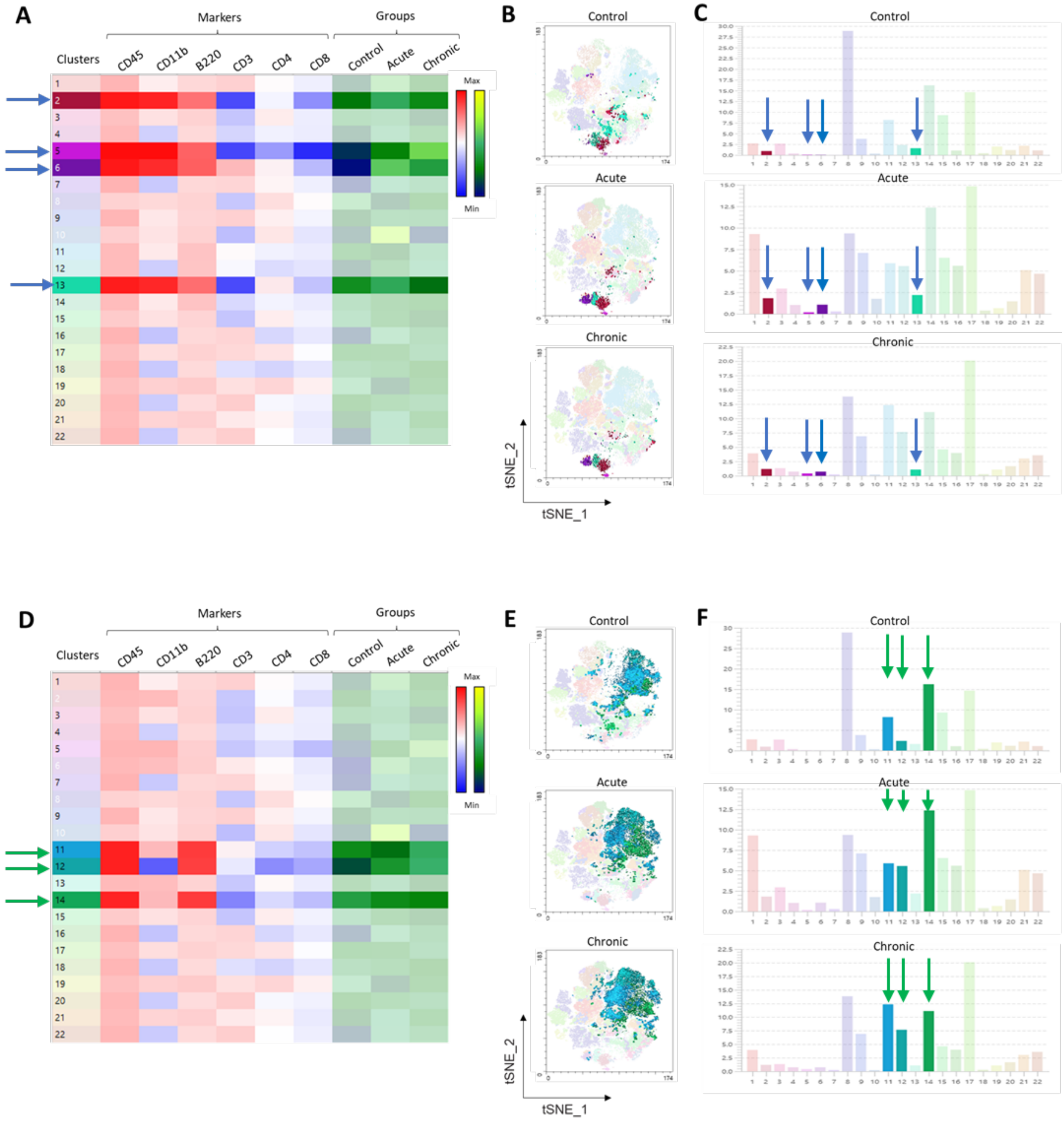

Supplementary Figure 2. Phenotypic analysis of myeloid cells and B cells via multiparameter spectral flow cytometry in the colonic lamina propria during acute and chronic *T. cruzi* infection. C57BL/6 mice were infected with 10^4 *T. cruzi* (TcCol-Nluc). Colonic lamina propria cells were isolated from uninfected (Control), acutely infected (Acute, 30 dpi) and chronically infected (Chronic, 90 dpi) and analysed by flow cytometry using the gating strategy shown in Supplemental Fig. 1. Myeloid cells; (A) heat maps for the cell surface markers expression indicated (Markers) and for control, acute and chronic colons (Groups). Highlighted are myeloid cells (CD 45^+, CD11b^+, clusters 2, 5, 6 and 13); (B) 2D tSNE map showing myeloid cells in Control, Acute and Chronic colons; (C) Percentage of myeloid cell clusters in each group. B

cells; (D) heat maps of B cells clusters (CD 45^+, $B220^+$, clusters 11, 12 and 14); (E) 2D tSNE map showing B cells; (F) Percentage of B cell clusters in each group.

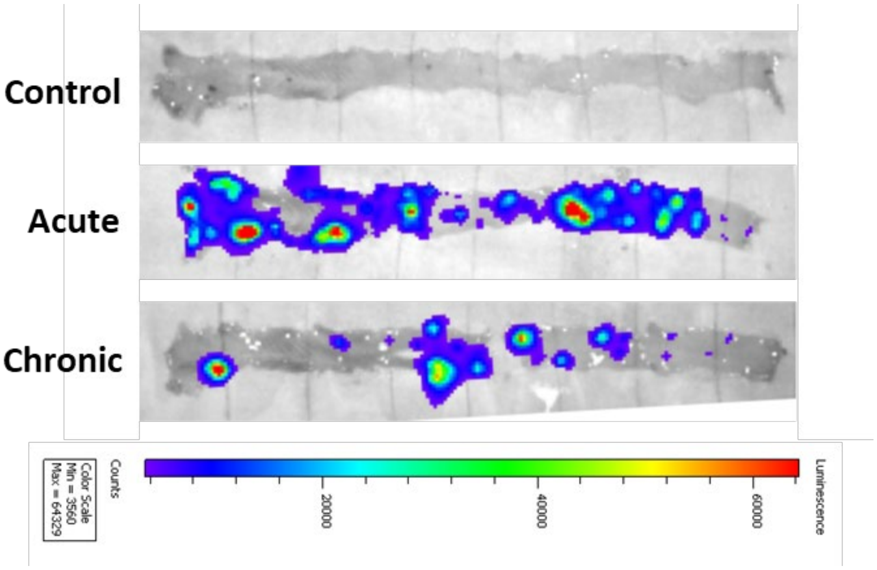

Supplementary Figure 3. Nanoluciferase expression of transgenic Tc-COL-NLuc-RFP parasites. C57BL/6 mice were infected with10^4 *T. cruzi* (TcCol-Nluc-RFP). Colons were isolated from uninfected (Control), acutely infected (Acute, 30 dpi) and chronically infected (Chronic, 90 dpi) and processed for *ex-vivo* imaging as described in the methods section. Log_{10} heat-map scales represent bioluminescence intensity (blue: low; red: high) expressed in p/sec/cm2/sr. Control: tissues from non-infected mice.

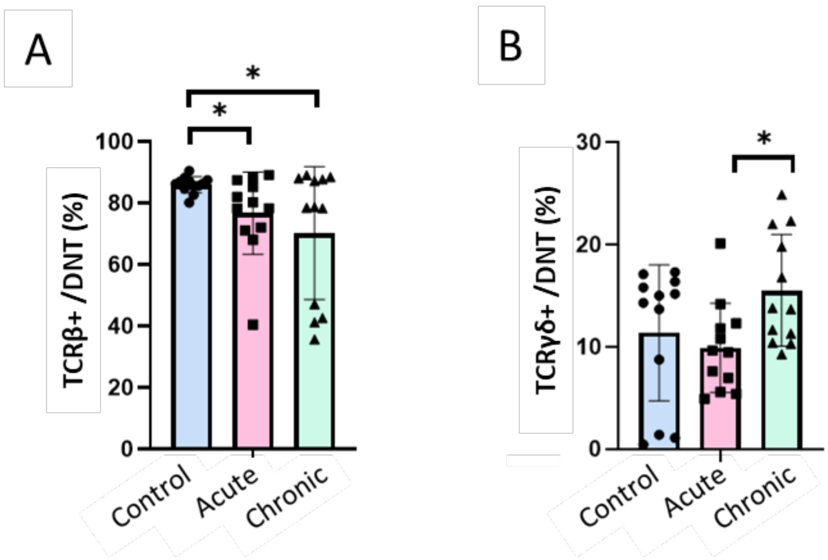

Supplementary Figure 4. TCR characterization of colonic DN T cells during *T. cruzi* infection. C57BL/6 mice were infected with 10^4 *T. cruzi* (TcCol-Nluc). Colonic lamina propria cells were isolated from uninfected (Control; blue), acutely infected (Acute, 30 dpi; pink) and chronically infected (Chronic, 90 dpi; green) mice and analyzed by flow cytometry. Cells were gated on single cells, live, $CD45^+$, $CD3^+$, $CD4^-$, $CD8^-$ events and subsequently analyzed for TCRβ and TCRγδ expression. **(A)** Quantification of $TCR\beta^+$ DN T cells as percentage of total DN T cells. **(B)** Quantification of $TCR\gamma\delta^+$ DN T cells as percentage of total DN T cells. Bars represent mean ± SD, and individual symbols denote values from single mice (n = 12 per group). Data represents the combination of 3 independent experiments. Statistical comparisons were made by an unpaired t test: $*p < 0.05$.

2. Supplementary Tables

Supplementary Table 1: Antibodies used in flow cytometry and immunofluorescence analyses

Panel	Catalog Number	Target/Marker/Antigen	Fluorochrome/Dye	Species Reactivity	Vendor	Clone	Isotype	RRID
1	103116	CD45	APC-Cy7	Mouse	BioLegend	30-F11	Rat IgG2b, κ	AB_312981
	563608	CD31	AlexaFluor647	Mouse	BD	390	IgG2a, κ	AB_2738313
	135923	PDGFR alpha	BV421	Mouse	BioLegend	APA5	Rat IgG2a, κ	AB_2814036
	147303	CDH1	PE	Human, Mouse, Dog, Pig	BioLegend	DECMA-1	Rat IgG1, κ	AB_2563039
2	103116	CD45	APC-Cy7	Mouse	BioLegend	30-F11	Rat IgG2b, κ	AB_312981
	100228	CD3	BV421	Mouse	BioLegend	17A2	Rat IgG2b, κ	AB_2562553
	100453	CD4	BV785	Mouse	BioLegend	GK1.5	Rat IgG2b, κ	AB_2565843
	100742	CD8	BV650	Mouse	BioLegend	53-6.7	Rat IgG2a, κ	AB_2563056
	101216	CD11b	PECy7	Human, Mouse, Baboon	BioLegend	M170	Rat IgG2b, κ	AB_312799
	103210	CD45R/B220	PECy5	Human, Mouse, Cat	BioLegend	RA3-6B2	Rat IgG2a, κ	AB_312995
3	553080	CD45	FITC	Mouse	BD	30-F11	IgG2b, κ	AB_394610
	100245	CD3	PE-Dazzle594	Mouse	BioLegend	17A2	Rat IgG2b, κ	AB_2565882
	100453	CD4	BV785	Mouse	BioLegend	GK1.5	Rat IgG2b, κ	AB_2565843
	100742	CD8	BV650	Mouse	BioLegend	53-6.7	Rat IgG2a, κ	AB_2563056
	130-120-168	CCR5	APC-Vio 770	Mouse	Miltenyi Biotec	REA354	Recombinant human IgG1	AB_2801755
	155930	CXCR3	PE-Fire 700	Mouse	BioLegend	S18001A	Rat IgG2b, κ	AB_3662195
	515407	Granzyme B	Pacific Blue	Human, Mouse, Rat	BioLegend	GB11	Mouse IgG1, κ	AB_2562195
	131217	CCR4	BV421	Mouse	BioLegend	2G12	Armenian Hamster IgG	AB_10933253
	505041	IL-10	BV711	Mouse	BioLegend	JES5-16E3	Rat IgG2b, κ	AB_3097268
	112705	IL10R	PE	Mouse	BioLegend	1B1.3a	Rat IgG1, κ	AB_313518
	130-123-842	TCR Beta	APC	Mouse	Miltenyi Biotec	H57-597	Recombinant human IgG1	AB_2905412
	118117	TCR g/d	PerCP-Cy5.5	Mouse	BioLegend	eBioGL-3	Armenian Hamster IgG	AB_10612572
4	AF554	CD4	Unconjugated	Mouse	R and D Systems	Polyclonal	Goat IgG	AB_355437
	NBP2-29475	CD8	Unconjugated	Human, Mouse, Porcine	Novus Biologicals	Polyclonal	Rabbit IgG	AB_2904552
	100209	CD3	AlexaFluor647	Mouse	BioLegend	17A2	Rat IgG2b, κ	AB_389323
	A11055	Secondary Antibody	AlexaFluor488	Goat	Thermo Fisher Scientific	Polyclonal	Donkey IgG	AB_2534102
	A10042	Secondary Antibody	AlexaFluor568	Rabbit	Thermo Fisher Scientific	Polyclonal	Donkey IgG	AB_2534017
